# The multiple faces of autoimmune Addison’s disease in children

**DOI:** 10.3389/fendo.2024.1411774

**Published:** 2024-09-16

**Authors:** Donatella Capalbo, Andrea Esposito, Valeria Gaeta, Paola Lorello, Sara Vasaturo, Raffaella Di Mase, Mariacarolina Salerno

**Affiliations:** ^1^ Pediatric Endocrinology Unit, Department of Translational Medical Sciences, University of Naples Federico II, Naples, Italy; ^2^ Department of Emergency, Santobono-Pausilipon Children’s Hospital, Naples, Italy; ^3^ Pediatric Endocrinology Unit, Department of Mother and Child, University Hospital Federico II, Naples, Italy

**Keywords:** primary adrenal insufficiency, autoimmune Addison’s disease, autoimmune polyendocrine syndromes, pediatric age, adrenal crises

## Abstract

Primary adrenal insufficiency (PAI) is a rare medical condition, characterized by a deficiency in adrenal hormones. Although rare, PAI is a life-threatening disease requiring prompt recognition and treatment. However, symptoms of PAI are often non-specific and diagnosis can be challenging, causing frequent diagnostic delays. In adults, autoimmunity is the most common cause of PAI in industrialized countries, whereas in children, the most frequent etiology is represented by congenital defects of steroidogenesis and, in particular, by congenital adrenal hyperplasia (CAH) due to 21-hydroxylase deficiency. A few recent case series from different countries have reported that autoimmunity is the second most common etiology of PAI in the pediatric age group. However, data on autoimmune PAI in children are still scant and the exact epidemiology, clinical manifestations, and long-term outcomes of this condition have yet to be defined. The scope of this review is to summarize the current knowledge on the etiology, presentation, and treatment of autoimmune PAI in childhood and to increase physicians’ awareness of the signs that should raise an early suspicion of this condition.

## Introduction

Primary adrenal insufficiency (PAI) is a rare medical condition, described for the first time by Thomas Addison in 1855, characterized by the impaired production of all hormones from the adrenal cortex. PAI is mainly characterized by a deficiency in glucocorticoids, but mineralocorticoid deficiency and androgen deficiency or excess may also occur (1). Although rare, PAI is a life-threatening disease requiring prompt recognition and treatment. However, diagnosis and management can be challenging, particularly in children, and there is a high chance of a greatly delayed diagnosis.

Several conditions, either congenital or acquired, can be responsible for PAI (2, 3). Autoimmune Addison’s disease (AAD) is the most common cause in adults in industrialized countries (4–7), whereas genetic defects are the most frequent etiology in children (2); indeed, congenital adrenal hyperplasia (CAH) due to 21-hydroxylase deficiency (21OH-D) represents the main cause of childhood-onset PAI. Nevertheless, among all possible causes of childhood-onset PAI, 21OH-D is the only condition that has been extensively characterized (8, 9); all other forms have not been adequately described, and the epidemiology, etiology, and long-term outcome of adrenal insufficiency in childhood still need to be well defined. Studies from the USA (10), Canada (11), and Finland (12) reported autoimmunity as the second most common cause of PAI in children following 21-OHD; accordingly, we confirm these data in a recent study describing the etiology of childhood-onset PAI in a large nationwide cohort from Italy (13). However, studies from Australia (14) and China (15) did not confirm this predominance.

It is well known that autoimmune adrenal insufficiency may develop in the context of several different conditions either in isolation or associated with other autoimmune disorders, and that clinical presentation differs depending on several factors, including the underlying disease. However, although many authors have described the epidemiology, presentation, and long-term outcomes of autoimmune forms in adults, data regarding the presentation and outcome of the autoimmune conditions responsible for AAD in children are still scant.

The aim of this review is to summarize the current knowledge on the etiology, presentation, and treatment of AAD in childhood.

## Case 1

A 13-year-old girl developed a loss of appetite associated with chronic fatigue and self-injurious behavior and was managed with psychological intervention. In the next 2 years, her condition progressively worsened; she continued to lose weight and started to present recurrent episodes of nausea, vomiting, and abdominal pain. Several gastrointestinal disorders were excluded. She eventually received a diagnosis of primary eating disorder and depression and started treatment with antidepressants without significant improvement. At the age of 15 years, she was hospitalized for acute deterioration in health status. At admission, she was dehydrated, tachycardic, and hypotensive (blood pressure, 90/50 mmHg). Biochemistry revealed the presence of metabolic acidosis, hyponatremia (sodium levels, 125 mmol/l, n.v. 135–145), hyperkalemia (potassium levels, 6.5 mmol/l, n.v. 3.5–5.0), and hypoglycemia (glucose levels, 49 mg/dl). Skin hyperpigmentation localized at gums and knuckles, was noted. Owing to the presence of hyperpigmentation and typical electrolyte abnormalities, adrenal function was assessed. Undetectable serum cortisol (<0.20 μg/dl), elevated adrenocorticotropic hormone (ACTH) concentration (1,237 pg/ml, n.v. 10–130), low aldosterone levels (<37 pg/ml, n.v. 40–432), and increased renin levels (500 pg/ml, n.v. 3.1–59.5) led to a diagnosis of PAI, and she was started on iv hydrocortisone treatment with a significant improvement in her clinical condition and electrolyte abnormalities. A diagnostic workup to assess the underlying etiology of the disease revealed the presence of adrenal autoantibodies, leading to a definitive diagnosis of AAD.

## Case 2

A boy with an uneventful history presented with protracted bloody diarrhea, nausea, and vomiting at the age of 9.9 years. Stool culture revealed the presence of *Campylobacter jejuni* and he received treatment with antibiotics. However, after a transient improvement, he continued to present recurrent vomiting (approximately two episodes/day) and developed severe constipation associated with long-lasting fatigue. Several investigations excluded gastrointestinal disorders. Eight months later, his conditions worsened, limiting his daily activities, and he had lost more than 5% of his body weight; therefore, he was referred to a pediatric hospital. Blood examination revealed hyponatremia (sodium, 121 mmol/l, n.v. 135–145) without other overt electrolyte abnormalities. At physical examination, the patient appeared to be slightly dehydrated and hypotensive; mild hyperpigmentation of the gums and labial mucosa was also noted, raising suspicion of AI. Adrenal function was therefore assessed and showed a significant increase in ACTH levels (877 pg/ml, n.v. 10–130), with low-normal levels of cortisol (7 μg/dl) and an increased concentration of plasma renin (2,398 pg/ml, n.v. 3.1–59.5). A standard-dose Synacthen test (SDSST) was performed because of equivocal levels of morning cortisol and definitively confirmed the diagnosis of PAI, revealing an insufficient peak of cortisol after stimulation (7.7 μg/dl). Investigations on the causes of primary adrenal failure, including steroid synthesis intermediates, very-long-chain fatty acid concentrations, and screening for infections, were negative. Adrenal cortex and 21-OH antibodies were positive, thus allowing a diagnosis of AAD.

## Case 3

A boy 10.7 years of age with Hashimoto’s thyroiditis was admitted at the emergency department after a few syncopal episodes occurring in the last 6 months. He complained about long-lasting fatigue and recurrent nausea. Blood tests were all within normal range except for slight hyponatremia (sodium levels ranging from 130 to 134 mmol/l, n.v. 135–145). No organic causes of nausea and hyponatremia were detected; therefore, the boy was discharged after iv hydration and an improvement in his general condition. Over the following 4 months, he continued to complain of weakness and nausea and started to present episodes of vomiting and walking difficulties. Physical examination revealed the presence of slight hypotension (blood pressure, 95/60 mmHg) and tachycardia (heart rate, 100 bpm). Moreover, slight skin hyperpigmentation localized at the gums, knuckles, and palmar creases was detected. Laboratory evaluation confirmed hyponatremia (128 mmol/l, n.v. 135–145) and normal levels of potassium (4.5 mmol/l, n.v. 3.5–5.0). Owing to the persistence of unexplained hyponatremia and hyperpigmentation in a patient with autoimmune thyroid disorder, adrenal function was assessed, revealing very low cortisol levels (3 µg/dl) associated with a moderate increase in ACTH (260 pg/ml, v.n.10–130) and renin concentrations (239 pg/ml, v.n. 0.9–13), leading to a diagnosis of PAI. Considering that he was affected by a comorbid known autoimmune condition, diagnostic workup started with an evaluation of adrenal autoantibodies, which was positive, confirming AAD.

## Autoimmune Addison’s disease

### Etiology

In AAD, the impaired production of adrenal hormones is due to the autoimmune destruction of the adrenal cortex, which typically first affects the zona glomerulosa and subsequently the zona fasciculata and reticularis. As for other autoimmune diseases, environmental triggers as viral infections, therapeutic agents, and mental health disorders are thought to initiate an autoimmune attack of the adrenal cortex in susceptible individuals (3). The effector cells of this autoimmune attack are thought to be CD8 and CD4 lymphocytes directed against 21-OH (3, 5, 16). Although autoimmune destruction of the adrenal cortex is due to cell-mediated immunity, AAD is characterized by the presence of non-pathogenic autoantibodies against the adrenal cortex, which are currently considered the gold standard for the diagnosis, monitoring, and prediction of AAD (3, 5, 16).

AAD can occur as an isolated condition, although in more than two-thirds of patients it is associated with other autoimmune disorders in the context of autoimmune polyglandular syndromes (APSs) (3, 7, 16, 17). APSs include a heterogeneous group of diseases sharing fundamental characteristics (18) (**Table 1**). Although there is no unanimous consensus on their classification, currently, APSs are mainly classified as APS type 1 (APS-1) and type 2 (APS-2) (19), and AAD represents the bridge between these two entities.

**Table 1:** The main features of autoimmune polyglandular syndromes (APSs).

	APS-1	APS-2
Prevalence	1:100,000 (variable)	1:20,000
Typical age at onset	Childhood	Adolescence-early adulthood
Inheritance	Monogenic	Polygenic
Genetic background	AIRE	HLA-DR3 and HLA-DR4 CTLA4 PTPN22
Main clinical features	Chronic mucocutaneous candidiasis Chronic hypoparathyroidism Autoimmune Addison’s disease	Autoimmune Addison’s disease Autoimmune thyroiditis Type 1 diabetes
Additional features	Hypergonadotropic hypogonadism Type 1 diabetes Autoimmune thyroiditis Autoimmune hypophysitis Alopecia Vitiligo Recurrent urticariod rash Nail dystrophy Autoimmune gastritis Autoimmune hepatitis Chronic diarrhea Constipation Malabsorption Autoimmune intestinal dysfunction	Hypergonadotropic hypogonadism Vitiligo Chronic atrophic gastritis Autoimmune hepatitis Celiac disease Myasthenia gravis Alopecia

APS-1, also named autoimmune polyendocrinopathy candidiasis ectodermal dystrophy (APECED), is a rare monogenic disease caused by mutations in the autoimmune regulator gene (*AIRE*) located on chromosome 21. Classically APS-1 was considered an autosomal recessive disease, but recently dominant negative mutations associated with milder autoimmune manifestations have been identified (19, 20). *AIRE* is expressed in medullary thymic epithelial cells and induces the expression of self-proteins expressed in other tissues, allowing their presentation to developing T cells and inducing apoptosis of self-reactive thymocytes (18–20). Moreover, AIRE promotes the generation of self-antigen-specific regulatory T cells that are involved in maintaining self-tolerance (18–20). Therefore, the absence of AIRE determines an impairment in deletion and in the peripheral control of autoreactive T cells with autoimmune attack against multiple tissues and organs (18, 19). In addition, reduced T-cell tolerance determines a dysregulation of B cells with a consequent production of autoantibodies directed against cytokines and tissue antigens that are considered specific markers of corresponding organ-specific autoimmune diseases (20, 21). Indeed, patients with APS-1 present multiple autoantibodies directed against tissue-specific antigens that often precede the onset of the corresponding clinical manifestation; among them, the most specific are autoantibodies against interferon-α and interferon-ω, which are almost invariably detected in affected subjects and have been proposed as a valid diagnostic aid (21, 22).

Despite its monogenic etiology, great variability in the spectrum and severity of APS-1 has been documented. A widely variable clinical picture has been observed even in patients carrying the same *AIRE* mutation, thus suggesting the influence of other environmental, immunological, or genetic factors on the expression of the disease (16, 19, 20, 23). An example are MHC alleles; studies on large cohorts of subjects have indeed demonstrated that patients with APS-1 carrying the DRB1*03 allele have a higher prevalence of AAD than those with different HLA haplotypes (24).

APS-1 is a rare disease and its prevalence is estimated at 1:100.000 and is higher in some particular populations, such as Iranian Jewish (1:9.000), Finland (1:25.000), Norway (1:90.000), Poland (1:129.000), and Ireland (1:130.000) (25, 26). APS-1 usually presents in childhood or in early adolescence (19, 27) and is clinically defined by the presence of at least two of the following conditions: chronic mucocutaneous candidiasis (CMC), chronic hypoparathyroidism (CH), and Addison’s disease. Usually, CMC is the first manifestation appearing before the age of 5 years, followed by CH before the age of 10 years and then AAD (28), although a precise chronological order of presentation of different components is not always present (20).

The onset of Addison in APS-1 patients generally occurs within the second decade of life, and the mean age varies between 11 and 16 years, depending on the clinical series (18, 26); however, earlier onset has also been documented; according to current literature, the earliest age of presentation of AAD in patients with APS-1 is 2 years (12, 26, 29, 30).

The prevalence of AAD in patients with APS-1 is approximately 65%, with no gender difference, but a high variability in different case series has been reported. In a recent Italian series evaluating 158 subjects, the prevalence increased with age (77.2% at the end of follow-up). The clinical phenotype of APS-1 is complex and also includes several other endocrine and non-endocrine components (19, 20, 27). Hypergonadotropic hypogonadism, type 1 diabetes, autoimmune thyroiditis, and hypophysitis are the most common endocrinopathies, and ectodermal involvement is characterized by alopecia, vitiligo, recurrent urticariod rash, or nail dystrophy (20, 28). Gastrointestinal manifestations represented by autoimmune gastritis and hepatitis, chronic diarrhea, constipation, malabsorption, and autoimmune intestinal dysfunction are also common (20, 28, 31). Finally, rare features such as autoimmune pneumonitis, inflammatory demyelinating polyneuropathy, tubular interstitial nephritis, keratitis, and asplenia have been described in these patients (20, 28).

APS-2 is a more common condition characterized by at least two of the following endocrinopathies: AAD, autoimmune thyroiditis, and type 1 diabetes (19, 27). APS-2 prevalence is estimated at 1:20.000, with a male-to-female ratio of 1:3 (19, 25, 27). In contrast to APS-1, which typically presents in childhood, the peak incidence of APS-2 is in the third and fourth decade of life and the number of children reported in the literature is still limited (32). APS-2 is a multifactorial and polygenic disorder with a complex heritability. Indeed, genetic studies on APS-2 patients showed that the same genetic variants are associated with an increased risk of several autoimmune diseases (19). These variants are mainly localized in genes regulating immune system function. Specifically, the HLA system has been considered to have an important role in genetic predisposition; indeed, the HLA-DR3 and HLA-DR4 alleles have been associated with an increased risk of APS-2, although the underlying mechanism is still not completely understood (16, 33). In addition, polymorphisms in other genes involved in the regulation of the immune system, such as *CTLA4* and *PTPN22*, have been reported to be risk factors. However, to date, there is no genetic or immunological diagnostic marker of APS-2 and the diagnosis only relies on the co-occurrence of typical autoimmune diseases.

Autoimmune thyroid disease represents the most frequent autoimmune endocrinopathy in APS-2, occurring in approximately 70% of patients; type 1 diabetes occurs in approximately 40–60% of patients, and is often the first manifestation, and ADD occurs in 40–50% of affected subjects (25). Other possible manifestations in the context of APS-2 are primary hypogonadism, vitiligo, chronic atrophic gastritis, hepatitis, celiac disease, myasthenia gravis, and alopecia (27).

APS-2 is an evolutive disorder, and a large time interval between first and second manifestations can occur (25). For example, it has been estimated that up to 50% of patients with AAD may develop another autoimmune disease throughout their lives (34). Relevant autoantibodies may be detectable years before the onset of AAD and thus testing for autoantibodies may be helpful in assessing disease risk (19).

As already mentioned, so far only a few patients with a diagnosis of APS-2 during childhood and adolescence have been described in detail (32, 35–49). Age at the presentation of APS-2 varied between 8 and 17 years, and the disease was only diagnosed before the age of 10 years in a few patients. In these pediatric reports, AAD was highly frequent and represented the most common manifestation of APS-2 in association with autoimmune thyroiditis; other manifestations variably associated were type 1 diabetes, celiac disease, vitiligo, and hypergonadotropic hypogonadism. The diagnosis was made through periodic screening in subjects with a pre-existing autoimmune manifestation or following a life-threatening event, such as adrenal crisis or diabetic ketoacidosis.

In addition, other patients with childhood-onset APS-2 have been reported in several cohorts of subjects with PAI from Italy (13, 50), Canada (11), the USA (10), and Finland (12), although clinical presentation or outcome have not been provided in detail (**Table 2**).

**Table 2:** The prevalence of autoimmune PAI, isolated Addison’s, and APSs in pediatric case series.

	Perry et al. (2005) (11)	Hiesh et al. (2011) (10)	Capalbo et al. (2021) (13)	Borchers et al. (2023) (12)
Country	Canada	USA	Italy	Finland
Study population (n)	29	42	121	42
AAD (%)	45%	55%	37%	67%
- Isolated AAD (%)	31%	73%	20%	47%
- APS-1 (%)	38%	7%	55%	28%
- APS-2 (%)	31%	20%	25%	25%

AAD, Autoimmune Addison’s disease; APS, autoimmune polyglandular syndrome.

Finally, isolated AAD also presents high heritability as APSs. So far, several genetic variants associated with an increased risk of isolated AAD have been described. As for APS-2, HLA-DR3 and HLA-DR4 alleles as well as polymorphisms in *CTLA4* and *PTPN22* have been associated with an increased risk of AAD but also variants in several genes (*BACH2*, *GATA3*, *LPP*, *IKZF4*, *SH2B3*, *CIITA*, *SULT1A2*, *CLEC16*, *MIC-A*, *MIC-B*, *NLRP1*, *SIGLEC5*, *LIME1*, and *UBASH3A)* have been reported to represent genetic risk factors (3, 16, 33, 51).

### Epidemiology

Only few case series on autoimmune PAI in children are available and the frequency of AAD in childhood still needs to be established. Overall, AAD prevalence can be influenced by the patient’s age (18), as well as by the underlying etiology and the ethnicity. So far, AAD has been reported as the most common cause of PAI in children after 21-OHD CAH by some authors. Indeed, data from US, Canadian, Italian, and Finnish studies have identified an autoimmune etiology in up to 67% of non-CAH PAI children (10–13). The prevalence of autoimmune PAI, as well as the prevalence of APSs in comparison with isolated Addison’s, varies on the basis of the population, as reported in **Table 2**.

### Presentation

**Figure 1:**
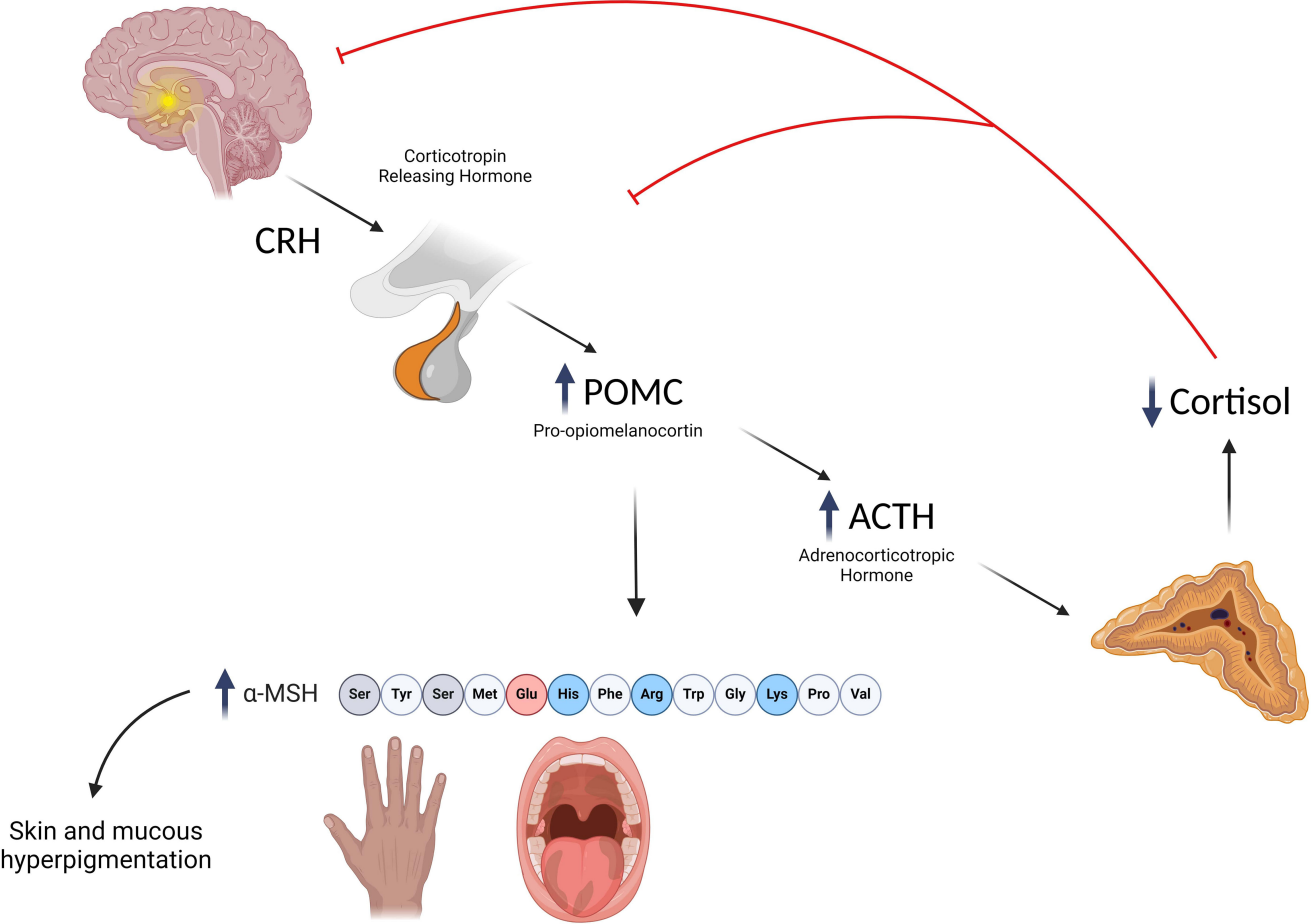
Mechanisms underlying the development of hyperpigmentation in primary adrenal insufficiency. Created with BioRender.com.

Autoimmune destruction of the adrenal cortex is a slow-progressive process; moreover, symptoms occur when up to 90% of adrenal cells are destroyed (5). As a consequence, AAD may typically remain subclinical for long periods before signs of AI become clinically evident (52). Even when adrenal insufficiency becomes manifest, clinical signs are often highly unspecific, including malaise, weakness, musculoskeletal pain, anorexia, reduced weight growth, orthostatic hypotension, salt craving, dizziness, abdominal pain, nausea, and vomiting (7), which can all be symptoms common to other different diseases. The vague nature of symptoms may often lead to misdiagnosis and cause considerable diagnostic delay. In a recent study, we reported that diagnostic delay is related to the underlying diagnosis being greater in children with AAD or X-ALD than in children with inherited monogenic conditions (13). Among all signs of PAI, hyperpigmentation is the most specific and represents the most remarkable clues for the diagnosis (3). A reduction in cortisol levels leads to the increased production of pro-opiomelanocortin (POMC), an ACTH precursor, and from its cleavage, alpha-melanocyte stimulating hormone (a-MSH) is also produced. Thus, PAI patients present increased levels of a-MSH, which acts on melanocytes favoring the production of eumelanin with a consequent increase in skin pigmentation, which is more evident in areas exposed to the sun (face, neck, back of the hand, and knuckles), normally hyperpigmented (nipple, scrotum, and labia), or subjected to friction and microtrauma (3) (**Figure 1**). Moreover, an increase in nevi, darkening of scars, and mucosae pigmentation are typical findings (53). Although being specific, hyperpigmentation may not be significant at the onset and therefore it is useful in identifying chronic but not acute adrenal insufficiency (2).

Typical laboratory findings of PAI include hyponatremia, hyperkalemia, and hypoglycemia. Hyponatremia is the most common alteration and even when isolated should raise the suspicion of AI (2, 3, 5, 13, 17, 53). Hypercalcemia, anemia, lymphocytosis, and eosinophilia can be rarely present (5, 53).

Owing to delays in the identification of symptoms and the variable length of time between presentation and definitive diagnosis, in many patients, a life-threatening adrenal crisis may be the first presentation of AAD (2, 3). Adrenal crisis is a serious condition characterized by weakness, muscular pain, vomiting, abdominal pain, dehydration, hypotension, impaired consciousness eventually associated with hyponatremia, hyperkalemia, and acidosis, which can lead to hypovolemic shock and death (54). However, clinical features at onset can be non-specific as well, and adrenal crisis can go unrecognized, especially in children (55). Common triggers for an adrenal crisis are acute stress as illness, infection, a surgical procedure, trauma, or severe psychological stress (7).

Finally, when AAD develops in the context of APSs, other autoimmune signs or diseases typical of APS type 1 or 2 (e.g., CMC, CH, and type 1 diabetes) may be present and in some cases may precede the onset of Addison’s, representing an important clue favoring an early diagnosis of PAI.

### Diagnostic approach

Once suspicion of adrenal insufficiency has been raised on the basis of clinical findings or history, an evaluation of cortisol and ACTH should be performed. A serum cortisol <5 µg/dl (<140 nmol/l) associated with increased ACTH (twice the upper normal limit) in the morning or during stress is diagnostic of PAI (1). In equivocal cases, a dynamic evaluation of adrenal function after SDSST can be performed to increase the sensitivity and specificity of basal hormonal measurement. Traditionally, a peak cortisol level of <18.0 µg/dl (500 nmol/l) after SDSST indicates adrenal insufficiency. However, this threshold is based on older serum assays having a high cross-reactivity with non-cortisol steroids. As the normal range of cortisol depends on the assay used, guidelines clearly indicate that checking the reference ranges of the laboratory is always recommended (1, 7). Indeed, newer more-specific immunoassays or LC-MS/MS may have lower thresholds for normal secretion and a recent study has suggested a new cortisol cutoff after SDSST of 14 (386 nmo/L) to 15 µg/dl (414 nmol/L) to define AI (depending on the specific assay) when using newer methods (56, 57), although specific ranges of normality still need to be established in children.

Recently, salivary cortisol has been proposed as valid alternative to serum cortisol (58), but standardization of the assays and accurate cutoffs are so far lacking (2).

High renin concentrations/activity and low aldosterone levels confirm mineralocorticoid deficiency (1). Of note, an increase in renin levels is an early marker of autoimmune adrenal insufficiency and precedes the alterations in ACTH and cortisol concentrations, and deserves particular attention. Indeed, autoimmune destruction typically first affects the zona glomerulosa, leading to mineralocortioid deficiency before glucocorticoid insufficiency (5).

**Figure 2:**
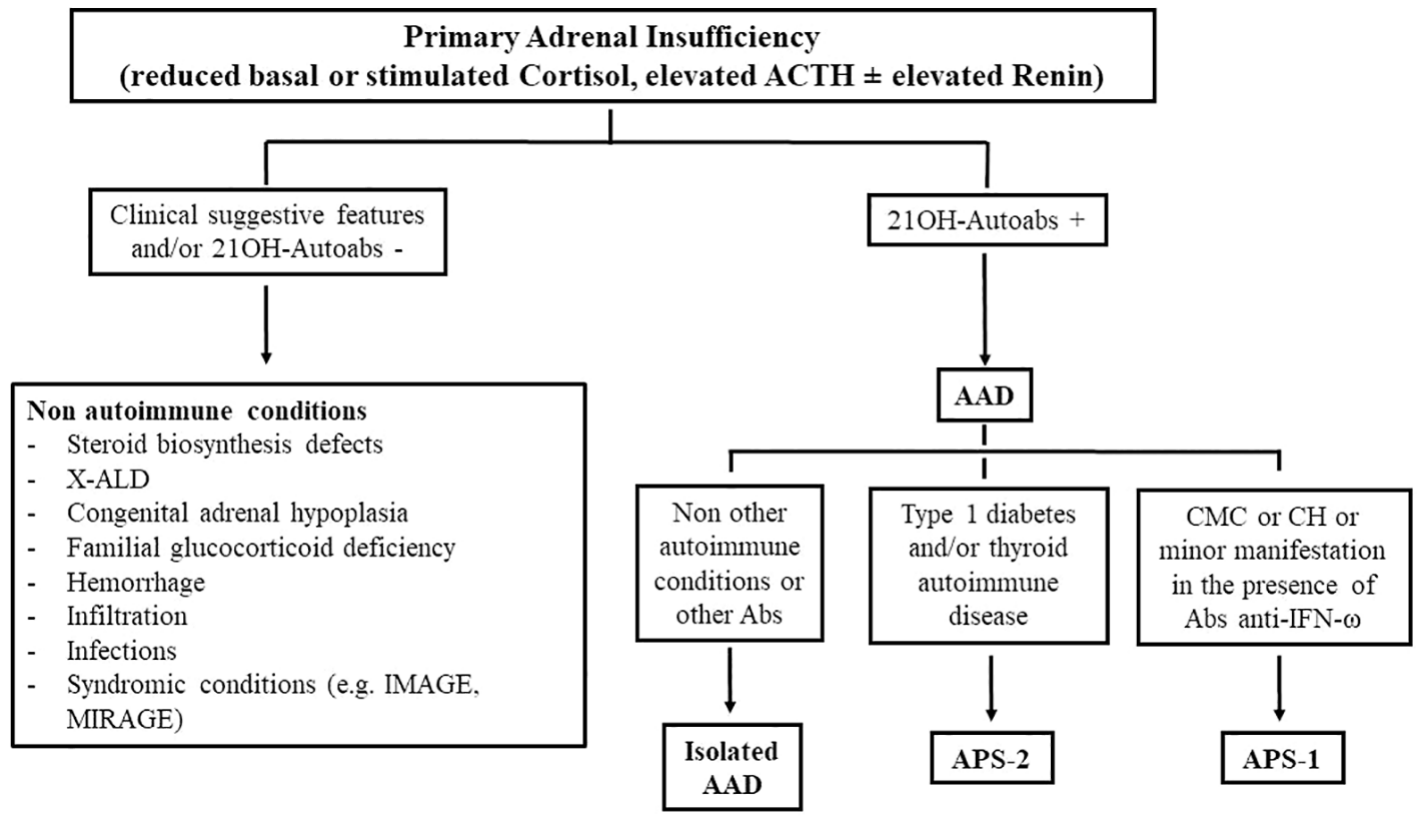
The diagnostic approach in autoimmune and non-autoimmune primary adrenal insufficiency. 21-OH, 21-hydroxylase; autoabs, autoantibodies; X-ALD, X-linked adrenoleukodystrophy; AAD, autoimmune Addison’s disease; CMC, chronic mucocutaneous candidiasis; CH, chronic hypoparathyroidism; IFN, interferon.

A stepwise diagnostic approach guided by age, the clinical presentation, and associated diseases is recommended (1, 2) (**Figure 2**). Autoimmune etiology is mainly supported by the presence of adrenal autoantibodies (17). These antibodies are directed against steroidogenic P450 autoantigens: 21-OH, 17α-OH, and cholesterol side-chain cleavage enzyme (17, 53, 59); 21-OH is typically expressed in all areas of the adrenal cortex, and 17α-OH and cholesterol side-chain cleavage enzyme are expressed in all steroid-producing cells, thus increasing the risk of primary autoimmune hypogonadism.

Autoantibodies targeting 21-OH are highly sensitive and they are currently considered markers of AAD, although they do not have a defined pathogenic role in the autoimmune destruction of the adrenal gland (53, 60). Indeed, 21-OH autoantibodies have been detected in 80–90% of subjects with PAI in cross-sectional studies after the exclusion of non-autoimmune conditions (61, 62). Moreover, so far, adrenal autoantibodies have been reported in very few patients without AAD and in particular in 1.4% of patients with non-adrenal autoimmune diseases, 5% of relatives of patients with AAD, and 0.6% of healthy individuals (63), thus confirming their high specificity.

The presence of adrenal antibodies might hint at a future risk of developing PAI as they may anticipate the clinical manifestation of AAD (17).

Indeed, it has been well established that the evolution of AAD presents different biochemical phases: stage 0, an isolated presence of autoantibodies with no impairment in adrenal function; stage 1, an isolated increase in renin concentrations/activity; stage 2, a reduced cortisol response after a stimulation test; stage 3, increased basal ACTH levels; and stage 4, reduced cortisol levels (5, 53) (**Table 3**). Therefore, owing to their predictive role, adrenal autoantibody testing in patients with pre-existing autoimmune diseases may help in identifying subjects at risk of developing AAD in a preclinical phase (64). In these patients, a periodical evaluation of adrenal function, including renin concentrations/activity, allows early diagnosis that can prevent a life-threatening adrenal crisis (27, 53). However, not all patients with positive adrenal autoantibodies will develop overt AI; the risk of progression toward clinical AAD is higher in patients with APS-1 (74–90%) than in patients with isolated AAD or APS-2 (0–44%) (64). In a recent Italian case series, although all APS-1 patients at stage 1 developed overt AAD, approximately 50% of APS-2 patients remained at stage 1 for a long period and 12% improved to stage 0; moreover, few APS-2 patients (2.5%) became negative during follow-up (64). However a lower threshold for the suspicion of AI in patients with pre-existing autoimmune morbidites is advisable. On the other hand, periodic screening for additional autoimmune diseases in patients diagnosed with AAD should be encouraged to correctly diagnose APSs (3, 7). For example, the association of AAD with autoimmune thyroiditis or type 1 diabetes is suggestive of APS-2 (19, 27), whereas the association with chronic mucocutaneous candidiasis or hypoparathyroidism suggests a diagnosis of APS-1, which needs be confirmed by an analysis of the *AIRE* gene (19, 20, 27) (**Figure 2**). With regard to the diagnosis of APS-1, recently diagnostic criteria seem to be expanding as it has been documented that, in some cases, minor manifestations may precede the onset of a classic dyad; among these manifestations, urticariod rash, enamel hypoplasia, and intestinal malabsorption were the most common (20). In these patients, screening for autoantibodies against interferon-ω or *AIRE* gene analysis should be performed to confirm diagnosis (19–22).

**Table 3:** Biochemical phases of the evolution of autoimmune Addison’s disease.

	Adrenal autoantibodies	Renin levels/activity	ACTH levels	Cortisol levels	Cortisol levels after stimulation	Symptoms
Stage 0	Present	Normal	Normal	Normal	Normal	Absent
Stage 1	Present	High	Normal	Normal	Normal	Absent
Stage 2	Present	High	Normal	Normal	Low	Absent
Stage 3	Present	High	High	Normal	Low	Absent
Stage 4	Present	High	High	Low	Low	Present

### Treatment and management

The treatment of autoimmune PAI mainly relies on hormone replacement therapy and treatment of complications. After PAI has been confirmed or in cases with highly suggestive severe symptoms, patients should begin replacement treatment with glucocorticoids and mineralocorticoids, without awaiting etiological confirmation (1). The adrenal cortex normally produces approximately 10 mg/m^2^ of cortisol every day with a specific circadian rhythm characterized by high levels in the early morning and low concentrations during the night. Oral hydrocortisone is the standard replacement treatment used in children with PAI. For AAD patients, hydrocortisone at a dose of 8–10 mg/m² per day divided into 3–4 doses is recommended, with a half to two-thirds of the total dose in the morning (1–3). A deficit in mineralocorticoids is almost invariably present in patients with AAD and fludrocortisone (9-alphafluorohydrocortisone) at a dosage of 0.05–0.2 mg/day in the morning should be added (1–3, 53). The treatment of adrenal crisis consists of the administration of iv saline and glucose and administration of hydrocortisone intravenously at a dose of 50–100 mg/m^2^ followed by 50–75 mg/m^2^ per day divided into four doses or as a continuous infusion (2, 3).

A key point in the management of AAD patients is represented by the prevention of adrenal crises, which still contribute to the mortality of PAI patients and are commonly triggered by infections, surgical procedures, intense physical activity, or treatment withdrawal (7, 53). The education of parents and patients plays a key role and should be repeated periodically during clinical evaluations (65). Indeed, all caregivers of PAI patients must understand the need to increase the glucocorticoid dose in case of illness, severe psychological/physical stress, or minor surgery (54) or administer intramuscular glucocorticoids when necessary; they must be also able to manage an incipient adrenal crisis (65). Furthermore, clinicians should recommend that patients wear an item indicating their condition in case of emergency (54, 66, 67).

In cases of APS-1 or APS-2, additional management consists of the adequate replacement of missing hormones and treatment of complications (19, 27).

It is suggested that children with AAD are seen by a pediatric endocrinologist or a healthcare provider with endocrine expertise (1). The suggested frequency of monitoring visits varies with age and associated morbidities or conditions. APS-1 patients are best followed by a multidisciplinary team led by an endocrinologist at a specialized center and with a minimum of two follow-up visits per year (19).

To date, no single biomarker for monitoring glucocorticoid treatment is available and the adequacy of treatment should be modulated on clinical evaluations, signs of overtreatment or undertreatment, general wellness, and growth (1, 3). Instead, salt craving, blood pressure, electrocyte levels, and renin concentrations/activity are used to assess mineralocorticoid treatment adequacy (2, 3, 53). As renin activity may lack accuracy, renin concentration is considered a more clinically relevant marker of the biochemical status and fludrocortisone dose (68, 69).

Physicians should always be aware that patients with AAD, in particular those affected with APSs, are at an increased risk for the development of another organ-specific autoimmunity, and periodic screening of autoantibodies or suggestive signs and symptoms of autoimmune diseases is advisable (3, 27). This screening includes an evaluation of thyroid function, diabetes mellitus, premature ovarian failure, celiac disease, and autoimmune gastritis, although the optimal frequency of screening is still not established and depends on the clinical evaluation as well as the diagnosis and complexity of the entire condition (7).

### Back to the patients

In the three described cases, Addison’s disease was diagnosed with a considerable delay as non-specific symptoms initially pointed to a wrong diagnosis. In all cases, hyperpigmentation helped in the recognition of PAI; however, hyperpigmentation is not significant at onset and is useful in identifying chronic but not acute adrenal insufficiency. Of note, each patient presented some specific clues that could have prompted an earlier diagnosis. In case 1, the diagnostic delay was largely related to her anchoring psychiatric diagnosis and she was correctly diagnosed only after adrenal crisis developed. This bias is common and PAI can be easily misdiagnosed with psychiatric diseases (70–73) as anorexia, weight loss, sleep disturbances, and behavior changes can be presenting symptoms of both diseases. Up until now, this patient has received a diagnosis of isolated AAD as autoimmune diagnostic work-up has not revealed any other autoantibody positivity or adjunctive manifestations during the follow-up.

In case 2, vomiting and abdominal pain dominated the clinical picture at onset thus leading to an erroneous suspicion of GI disorder. Indeed, gastrointestinal symptoms are common in the early stages of adrenal insufficiency (40, 74–77); in particular, nausea progressing to vomiting is a typical presentation of the disease. In this particular case, the index sign was the combination of vomiting, hyponatremia, and hyperpigmentation, which should have prompted a suspicion of AI. Although no other major sign was present, the early onset of the disease and the presence of specific signs as alternating constipation/diarrhea, raised a suspicion of APS-1 and the patient was tested for autoantibodies against interferon-ω, which was positive. *AIRE* gene analysis confirmed the diagnosis, revealing causative homozygous mutations (c.47C>T) of the gene. No other components of the disease have developed up to his last follow-up at the age of 11.5 years. The patient is currently on hydrocortisone and fludrocortisone replacement treatment. This case confirms the huge heterogeneity in the presentation of APECED, the need for high awareness, even in the absence of a classic triad, and the importance of an increased alert for adrenal insufficiency in case of unexplained GI symptoms (20, 28, 31).

Finally, in case 3, the presenting sign was characterized by isolated mild hyponatremia. Hyponatremia is indeed the most common laboratory finding in PAI (13), even in the absence of hyperkalemia. In this case, the pre-existing autoimmune thyroiditis hinted at a diagnosis and association of AAD, and thyroiditis led to a diagnosis of APS-2. During the follow-up, no adjunctive autoimmune diseases or signs were diagnosed. This history highlights how unexplained protracted hyponatremia should raise the suspicion of adrenal insufficiency, even in the absence of hyperkalemia or other specific signs (13), and points out the importance of an increased awareness for AI in children with other pre-existing autoimmune manifestations.

## Conclusion

AAD is an important cause of PAI in children, either in isolation or in the context of APSs. Clinical and biochemical signs are evident only when most adrenal function is impaired and are often non-specific, thus pointing to a wrong diagnosis, which puts patients at risk of a life-threatening adrenal crisis. Autoimmune PAI in children is associated with a greater diagnostic delay than inherited genetic conditions. Therefore, it is important to increase physicians’ awareness on signs that should raise an early suspicion of adrenal insufficiency, such as unexplained weakness, gastrointestinal or psychiatric disorders, or hyponatremia. An autoimmune condition should be suspected in all those subjects with adrenal insufficiency and without specific signs related to other genetic/syndromic disease, at any age. Of note, a low threshold for suspicion should also be kept in patients with known pre-existing autoimmune conditions. In these cases, the screening of adrenal autoantibodies is helpful in identifying at-risk subjects who deserve careful adrenal function monitoring.

The management of AAD patients should include a program of adrenal crisis prevention through the continuous education of patients and families in managing stressful events. Studies on large cohorts of children with autoimmune PAI are needed to increase the current knowledge on the presentation, natural history, and long-term outcomes of the disease in childhood and to improve the early recognition and treatment of the disease.
